# Contrast-enhanced mammography for breast cancer detection and diagnosis with high concentration iodinated contrast medium

**DOI:** 10.1186/s13244-025-01994-8

**Published:** 2025-06-14

**Authors:** Federica Pediconi, Annarita Speranza, Giuliana Moffa, Roberto Maroncelli, Sara Coppola, Francesca Galati, Claudia Bernardi, Giacomo Maccagno, Dominga Pugliese, Carlo Catalano, Andrea Laghi, Veronica Rizzo

**Affiliations:** 1 Department of Radiological, Oncological and Pathological Sciences, Sapienza, University of Rome, Policlinico “Umberto I”, Rome, Italy; 2 Department of Surgical and Medical Sciences and Translational Medicine, Sapienza, University of Rome, Sant’Andrea University Hospital, Rome, Italy

**Keywords:** CEM, Iodinated contrast media, High iodine concentration, Diagnostic performance, Breast cancer

## Abstract

**Objectives:**

We assessed the diagnostic performance of contrast-enhanced mammography (CEM) using a high-concentration iodinated contrast medium (HCCM, 400 mgI/mL) to determine whether the reduced iodine dose and increased iodine delivery rate (IDR) achieved might offer a more sustainable alternative to CEM performed with lower iodine concentrations.

**Methods:**

This two-center retrospective study included 205 patients who underwent CEM between March 2021 and February 2022. Patients were injected with HCCM at 1.0 mL/kg bodyweight at an IDR of 1.2 gL/s. Standard cranio-caudal and mediolateral-oblique views were acquired. Images were reviewed independently by two experienced radiologists who were blinded to patient clinical and imaging information. Diagnostic performance, including sensitivity, specificity, and accuracy, was assessed based on histological or long-term imaging follow-up as the reference standard.

**Results:**

Among the 205 patients, 149 (72.7%) had malignant lesions, and 56 (27.3%) had benign findings. The sensitivity and specificity of CEM were 96–97% and 84–87.5%, respectively, with an overall accuracy of 93–95%. The IDR achieved with HCCM resulted in excellent contrast enhancement, particularly in patients with aggressive malignancies. ROC analysis confirmed the good diagnostic performance, with AUC values of 0.90–0.92. Compared to conventional mammography and ultrasound, CEM demonstrated significantly higher diagnostic accuracy, especially in patients with dense breast tissue.

**Conclusions:**

CEM with HCCM provides excellent diagnostic performance, achieving high sensitivity and specificity while allowing for a reduced iodine dose and increased IDR. This approach may offer a more sustainable alternative to conventional contrast media without compromising diagnostic accuracy, particularly for the detection and characterization of aggressive breast lesions.

**Critical relevance statement:**

This study demonstrates that reducing the volume of injected contrast media while increasing iodine concentration maintains the diagnostic benefits of CEM, further supporting its potential to improve early cancer detection, thereby advancing clinical radiology practices and optimizing screening strategies for women with dense breasts.

**Key Points:**

- Currently, CEM protocols utilize a variety of iodine concentrations and flow rates.
- CEM with high-concentration contrast (400 mgI/mL) achieved 96% sensitivity and 87.5% specificity.
- High-concentration contrast in CEM improves early detection of aggressive breast cancers.

**Graphical Abstract:**

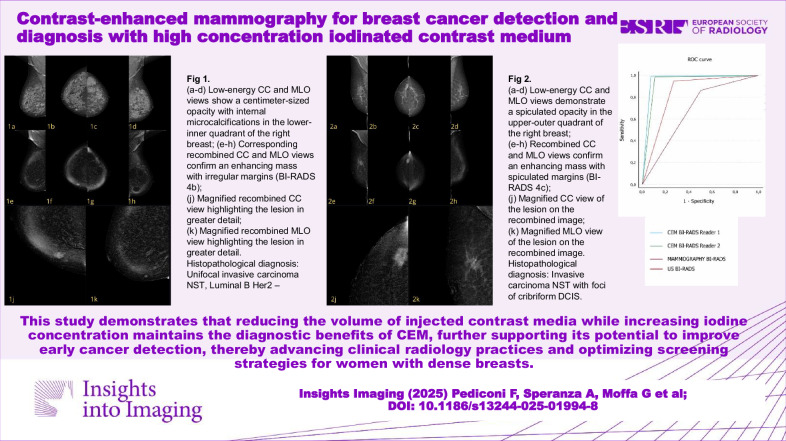

## Introduction

Contrast-enhanced mammography (CEM) is an increasingly used breast imaging technique for breast cancer detection and diagnosis [1–6]. Unlike conventional 2D digital mammography (DM) and 3D digital breast tomosynthesis techniques, CEM offers the possibility to acquire both morphologic and physiologic information similar to that attainable with contrast-enhanced breast magnetic resonance imaging (MRI). In recombining high-energy mammographic images acquired after the intravenous administration of an iodinated contrast medium (CM), CEM facilitates better detection of fast-growing aggressive lesions and improved differentiation among lesions with different proliferative and metastatic potential, by providing functional information from CM uptake as a proxy of malignant neoangiogenesis [2, 3]. In this regard, numerous studies and meta-analyses have demonstrated comparable diagnostic performance for CEM and contrast-enhanced MRI [7–13], often with sensitivity and specificity values exceeding 90% for the detection and characterization of malignancy [13]. Moreover, potential benefits of CEM are that it is more accessible, affordable, and potentially better tolerated than contrast-enhanced MRI, albeit with the necessary requirement for ionizing radiation exposure [14].

Although many studies have looked to evaluate the diagnostic performance of CEM, comparatively few have focused on the CEM technique and specifically on the dose, concentration, and flow rate of the iodinated CM used for the examination. Some studies simply do not provide information about the type of iodinated CM used [9] while others state that a low osmolar non-ionic CM should be administered at 1.5 mL/kg (for a maximum of 150 mL) and at a rate of 2–3 mL/s, without specifying the iodine concentration of the CM [2–4] but rather stating that the typical CM utilized should have iodine concentrations ranging from 300 mgI/mL to 370 mgI/mL [2, 3]. Clearly, for a given patient of 75 kg, 112.5 mL (i.e., 1.5 mL/kg × 75 kg) of a CM containing 370 mgI/mL would lead to a considerably higher iodine dose than 112.5 mL of a CM containing 300 mgI/mL administered at the same rate (41.63 g iodine vs 33.75 g iodine). Given recent shortages in iodinated CM availability [15, 16] and increasing concern over the potential harmful environmental impact of iodinated CM [17, 18], considerable attention now focuses on a more sustainable use of iodinated CM [19] and particularly on opportunities for iodine dose lowering.

A benefit of a higher iodine concentration is that more iodine is administered per unit of volume. For standard injection protocols using a flow rate of 3 mL/s, a CM containing 400 mgI/mL will give an iodine delivery rate (IDR) of 1.2 gI/s. In contrast, CM containing 300–370 mgI/mL will give lower IDRs of 0.9–1.1 gI/s. This means that with high concentration contrast medium (HCCM), more iodine will reach blood vessels in a given time, which may be advantageous when assessing potential malignant neoangiogenesis. It is additionally noteworthy that a higher iodine concentration also means that the overall volume of CM administered during each examination can be reduced for a given iodine dose. Potential advantages of a reduced administration volume are reduced contrast waste and reduced cardiac preload, which potentially improves patient tolerability during contrast injection [20]. Importantly, as noted elsewhere [21], a higher iodine concentration is not associated with an increased risk of nephrotoxicity.

The fact that more iodine is administered per unit of volume with HCCM means that the volume administered per kg of patient can be reduced while maintaining contrast enhancement. The aim of this study was to determine the diagnostic potential of CEM when performed with HCCM (400 mgI/mL; Iomeprol-400; Bracco) at a lower overall iodine dose and a higher IDR than typically attainable with CM containing lower concentrations of iodine (300–370 mgI/mL), to determine whether HCCM potentially offers a more sustainable solution to CM use in CEM. Specifically, we determined the diagnostic performance of CEM performed with 1.0 mL/kg of Iomeprol-400 (400 mgI/mL) administered at a rate of 3 mL/s for an IDR of 1.2 gI/s and compared the results with literature reports of CEM studies performed with lower concentrated CM administered using “standard” protocols (1.5 mL/kg at a rate of 2–3 mL/s).

## Methods

### Study design and population

This two-center retrospective study was conducted according to Good Clinical Practice guidelines. The evaluation included patients referred for CEM at one of the following two Italian imaging departments: Policlinico Umberto I, Rome (Center 1), and Ospedale Sant’Andrea, Rome (Center 2). Approval for the retrospective assessment of images was obtained by the Institutional Review Boards of both centers. The requirement for informed consent was waived because of the retrospective nature of the study.

All women referred for breast imaging at either center between March 2021 and February 2022, who had undergone initial conventional DM and ultrasound (US) examinations and who were eligible for CEM as a second-level diagnostic examination, were included in the study. CEM was used as a problem solver when conventional imaging results were contradictory or inconclusive, for example, when abnormalities detected at previous DM were not clarified by US examination. Additionally, CEM was used for preoperative staging, particularly in cases of suspicious multifocal or multicentric disease, and for monitoring the response to treatment. CEM was not performed in patients who were pregnant or suspected of being pregnant, were lactating, had a history of allergic reaction to iodinated contrast agents, or had impaired renal function based on the latest guidelines of the European Society of Urogenital Radiology (ESUR) [22]. Patients who underwent CEM at either of the two centers within the study period were excluded from the study if they had a history of breast cancer or recurrent disease, were undergoing neoadjuvant therapy or any other cancer treatment, had breast implants, or had undergone core needle biopsy (CNB) or vacuum-assisted biopsy (VAB) within 14 days prior to the CEM examination. Finally, patients with incomplete clinical/histological data, or whose CEM examination was incomplete, were also excluded from the study.

All data were acquired anonymously from Institutional medical records and collected using Excel 2016 (Microsoft Corp.).

### Imaging technique and protocol

All CEM examinations were performed on dedicated low-dose DM units that were capable of performing full-field 2D DM and CEM (Giotto Class; IMS Giotto, for Center 1, and Senographe Essential; GE Healthcare, for Center 2). Written informed consent was obtained from all patients prior to initiating the CEM examination.

Similar protocols were utilized at both centers, involving the sequential acquisition of two standard cranio-caudal and two mediolateral-oblique views (early and late, respectively) starting from the breast with the suspicious finding. Image acquisition began 2 min after the intravenous injection of iodinated CM (Iomeprol-400; 400 mgI/mL) at 1.0 mL/kg bodyweight and at a rate of 3 mL/s, followed by a 20-mL saline flush at the same rate. The administered volume (1.0 mL/kg) results in a dose of 400 mgI/kg bodyweight administered at an IDR of 1.2 gI/s. A low-energy exposure (22–35 kVp) and a high-energy exposure (40–49 kVp) were performed serially for each view. The total acquisition time was approximately 6 min, resulting in an overall examination time of approximately 8 min.

### Image evaluation and interpretation

The American College of Radiology (ACR) breast imaging reporting and data system (BI-RADS) categories assigned to findings from DM and US examinations of the patients enrolled in the study were collected from medical records. Two experienced breast imaging radiologists independently evaluated all the anonymized CEM examinations from both centers, using a dedicated workstation equipped with an integrated software (Raffaello, IMS Giotto for Center 1 and AWS:52.21.3 Essential Senographe Essential; GE Healthcare for Center 2) and two dedicated 5-Megapixel diagnostic LED monitors (GX570; Eizo) for both centers. Both readers were blinded to the results of previous breast imaging examinations and to all patient clinical and pathological information. CEM image assessment was performed separately to reduce possible bias, using both low-energy and recombined images.

Breast composition, defined as ACR BI-RADS categories A-D, and background parenchymal enhancement (BPE), defined as categories 1-4, were evaluated visually and classified according to the current ACR BI-RADS lexicon [23]. Each finding (including calcifications) was measured and classified based on the CEM supplement to the 2013 ACR BI-RADS lexicon for mammography [24], and a final BI-RADS assessment category was assigned. BI-RADS 1–3 findings were considered benign, while BI-RADS 4 and 5 findings were considered malignant. The assignation of a BI-RADS category 0 was not allowed.

Histology results from CNB, VAB, or surgery (when available), or findings from follow-up diagnostic imaging examinations with CEM or contrast-enhanced MRI after ≥ 12 months, were considered the standard of reference for the determination of diagnostic performance.

### Histological analysis

Biopsy and surgical specimens were evaluated at each center by dedicated pathologists following standardized protocols. Lesions were classified according to the fourth edition of the World Health Organization Classification [25]. Malignant primary breast tumors were subsequently tested for immunohistochemistry using mouse monoclonal antibodies anti-estrogen receptor (ER) alpha (6F11; Novocastra Laboratories Ltd., Newcastle upon Tyne, UK) and anti-progesterone receptor (PgR-312; Novocastra Laboratories Ltd., Newcastle upon Tyne, UK). A semiquantitative immunohistochemical assay (HercepTest; Dako Agilent) was employed to evaluate the HER2 status; equivocal results were further evaluated by means of fluorescence in situ hybridization for HER2 gene amplification, according to the 2013 American Society of Clinical Oncology/College of American Pathologists guidelines [26]. Tumor proliferation index was determined using anti-Ki-67 monoclonal antibody MM1 (Novocastra Laboratories Ltd.). Based on the results of immunohistochemistry, primary malignant lesions were classified according to the 2013 St. Gallen Consensus Conference [27] as: luminal A-like, luminal B-like HER2-negative, luminal B-like HER2-positive, HER2-positive, or triple-negative.

### Statistical analysis

All statistical analyses and graphical representations were performed using IBM SPSS Statistics, version 28 (IBM). Sensitivity, specificity, positive predictive value (PPV), negative predictive value (NPV), and overall accuracy were calculated for each reader.

The receiver operating characteristic (ROC) curve and the corresponding area under the curve (AUC) value were used to estimate the overall diagnostic performance for both readers and all imaging techniques. Cohen’s Kappa coefficient was used to determine inter-reader agreement for lesion detection and classification based on the ACR BI-RADS lexicon [23]. Spearman’s correlation was used to highlight potential correlations between ordinal variables.

## Results

**Fig. 1:**
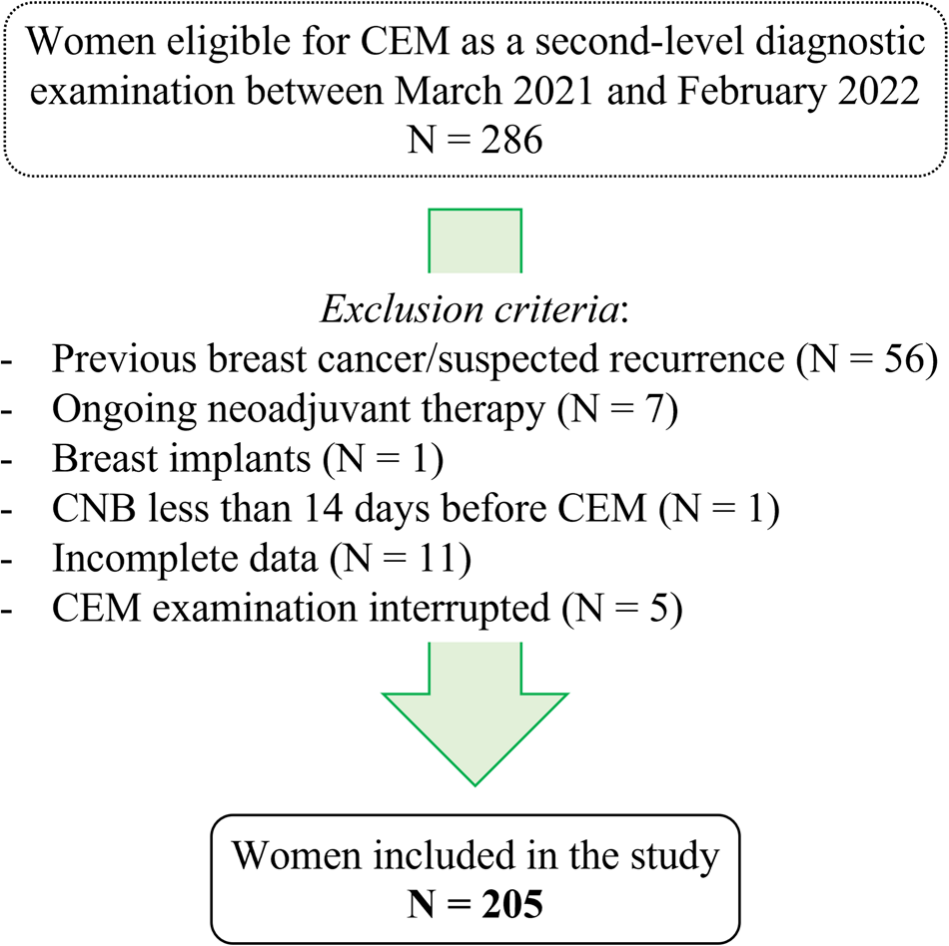
Selection flowchart showing inclusion and exclusion criteria of the study

A total of 286 women were initially screened for inclusion in the study. Of these, 205 were considered eligible for further analysis (mean age: 58 ± 12.3 years; range: 29–85 years). Of the 81 ineligible patients, 56 were excluded because of previous breast cancer or suspected recurrence, 7 were undergoing neoadjuvant therapy, 1 had breast implants, 1 had undergone CNB less than 14 days before the CEM examination, and 11 had incomplete data. The remaining 5 patients were excluded because the CEM examination was interrupted before being completed. The selection algorithm is summarized in Fig. 1.

**Fig. 2:**
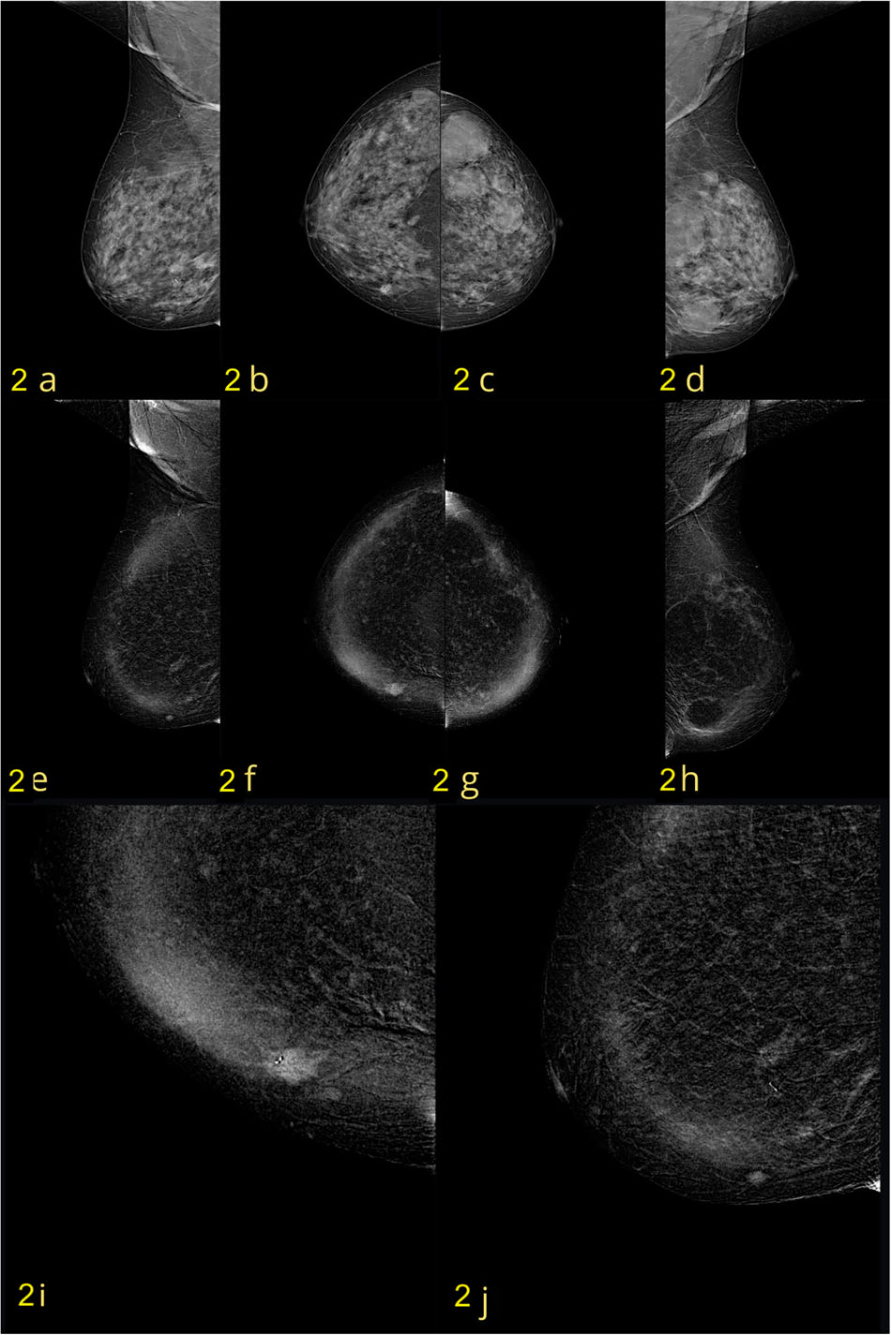
A case of a 50-year-old woman, classified as ACR BI-RADS D. **a**–**d** Low-energy CC and MLO views show a centimeter-sized opacity with internal microcalcifications in the lower-inner quadrant of the right breast. **e**–**h** Corresponding recombined CC and MLO views confirm an enhancing mass with irregular margins (BI-RADS 4b). **i** Magnified the recombined CC view, highlighting the lesion in greater detail. **j** Magnified recombined MLO view highlighting the lesion in greater detail. Histopathological diagnosis: unifocal invasive carcinoma NST, Luminal B Her2–

The 205 eligible patients included 149 (72.7%) in whom malignant lesions were identified, and 56 (27.3%) in whom benign lesions were identified. The 149 malignant lesions identified comprised invasive cancer of no special type (NST; *n* = 114 [55.6%]; Fig. 2), ductal carcinoma in situ (DCIS; *n* = 13; [6.3%]), invasive lobular carcinoma (ILC; *n* = 21; [10.3%]), and metastasis (*n* = 1 [0.5%]). Histological confirmation was based on CNB, VAB, surgery, and ≥ 12-month follow-up in 52, 34, 85, and 34 cases, respectively. Analysis of molecular subtypes was performed for all malignant lesions except the solitary case of breast metastasis (i.e., for 148/149 malignant lesions). Our data demonstrate that CEM with HCCM consistently outperforms conventional DM in differentiating molecular subtypes (Table 1).

**Table 1:** Classification of molecular subtypes of breast cancer according to histological subtype and assessment of sensitivity based on different diagnostic tests and readers

	NST (114)	ILC (21)	DCIS (13)	Mammography sensitivity	US sensitivity	CEM Reader 1 sensitivity	CEM Reader 2 sensitivity
Luminal A (58 patients)	45	8	5	86.2	94.8	98.3	96.6
Luminal B HER2-(60 patients)	43	13	4	86.7	93.3	98.3	96.7
Luminal B HER2 + (5 patients)	3	0	2	80	100	80	80
HeER + (9 patients)	8	0	1	88.9	88.9	100	100
TN (16 patients)	15	0	1	68.8	100	93.8	93.8

**Fig. 3:**
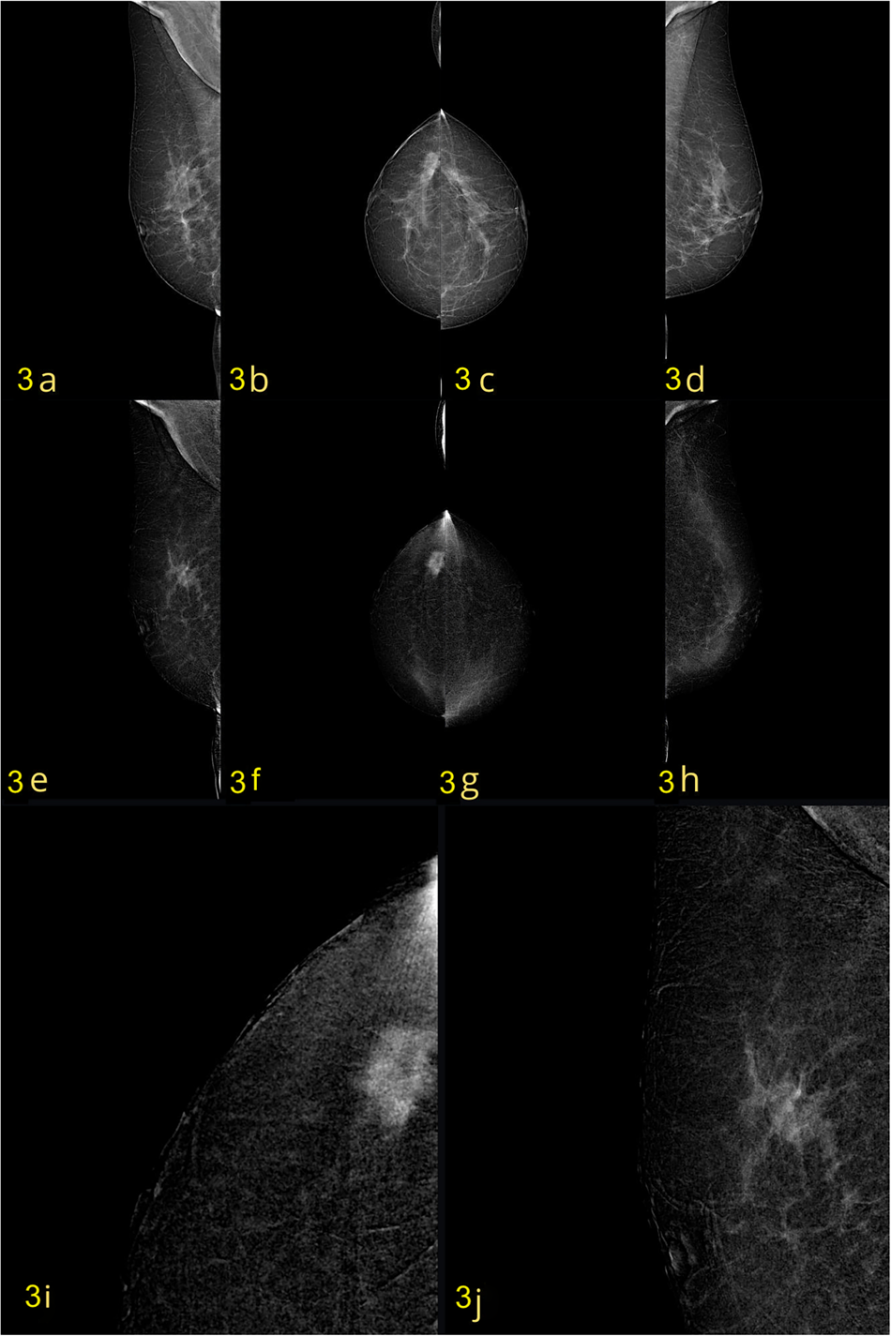
A case of a 49-year-old woman, classified as ACR BI-RADS B. **a**–**d** Low-energy CC and MLO views demonstrate a spiculated opacity in the upper-outer quadrant of the right breast. **e**–**h** Recombined CC and MLO views confirm an enhancing mass with spiculated margins (BI-RADS 4c). **i** Magnified the CC view of the lesion on the recombined image. **j** Magnified MLO view of the lesion on the recombined image. Histopathological diagnosis: invasive carcinoma NST with foci of cribriform DCIS

**Fig. 4:**
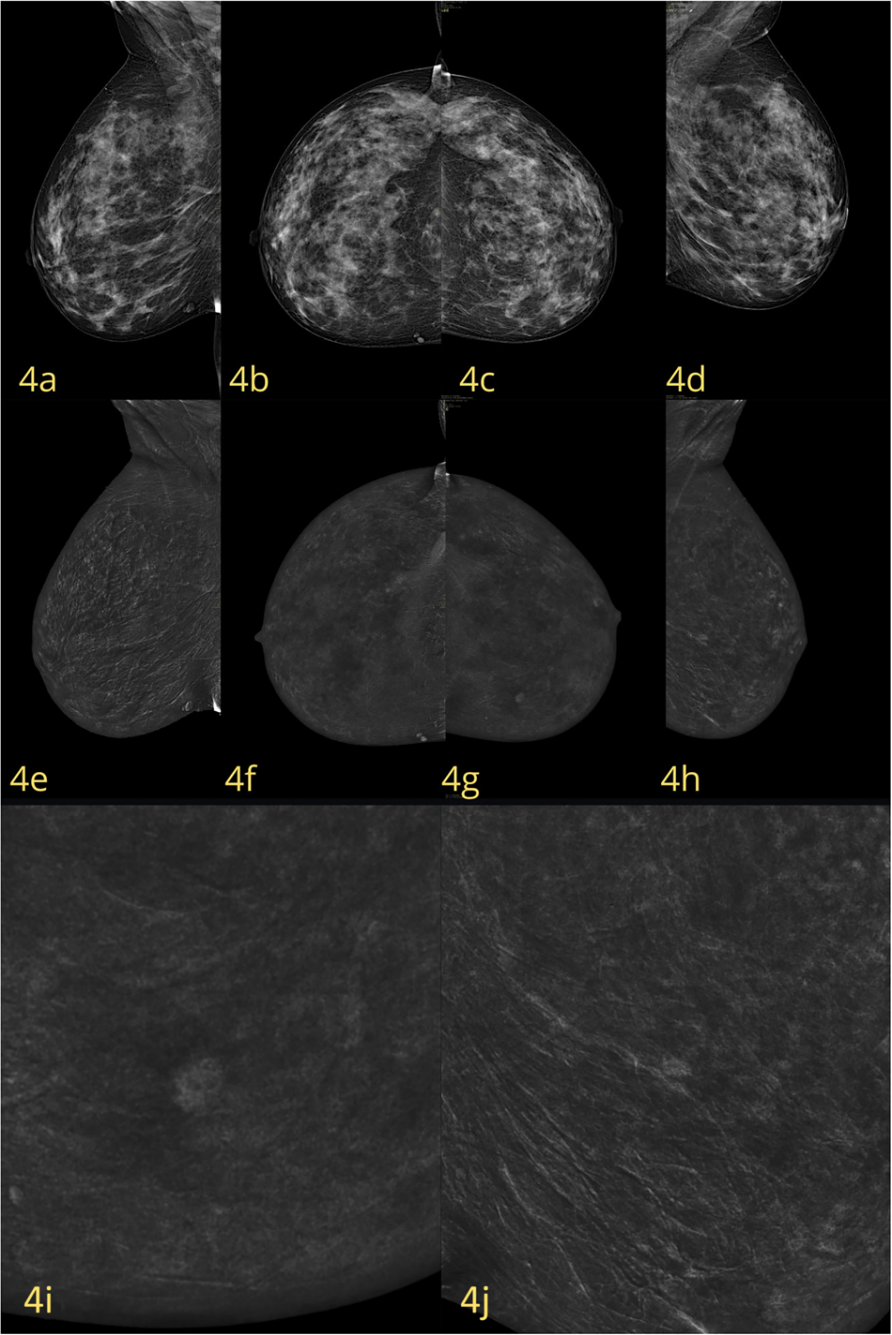
A case of a 42-year-old woman, classified as ACR BI-RADS C. **a**–**d** Low-energy CC and MLO views reveal a dense breast, with no evident mass, asymmetry, or parenchymal distortion; CEM was suggested for the presence of amorphous microcalcification in the upper outer quadrant of the left breast (BIRADS 4a). **e**–**h** Recombined CC and MLO views show no enhancing lesions in the upper outer quadrant (in the site of microcalcification), but the presence of an enhancing area of 8 mm, with uneven margins, between the lower quadrants. **i** Magnified the CC view of the lesion on the recombined image. **j** Magnified MLO view of the lesion on the recombined image. CEM-guided biopsy was performed, and histopathological diagnosis showed an invasive carcinoma NST, Luminal A

The characteristics of the evaluated women in terms of breast composition and BPE are reported in Table 2. The majority of patients (85.4% for both readers) had breast composition categorized as either B (scattered areas of fibroglandular density; Fig. 3) or C (heterogeneously dense; Fig. 4). Most patients (166/205 [81%] and 168/205 [82%] for readers 1 and 2, respectively) exhibited minimal to mild BPE (ACR BI-RADS categories 1 or 2).

**Table 2:** Classification of breast compositions and BPE according to Reader 1 and Reader 2

	Reader 1 patients (%)	Reader 2 patients (%)
Breast composition (ACR BI-RADS)		
A	13 (6.3)	12 (5.8)
B	86 (42)	86 (42)
C	89 (43.4)	89 (43.4)
D	17 (8.3)	18 (8.8)
BPE		
1	114 (55.6)	109 (53.2)
2	52 (25.4)	59 (28.8)
3	31 (15.1)	30 (14.6)
4	8 (3.9)	7 (3.4)

**Fig. 5:**
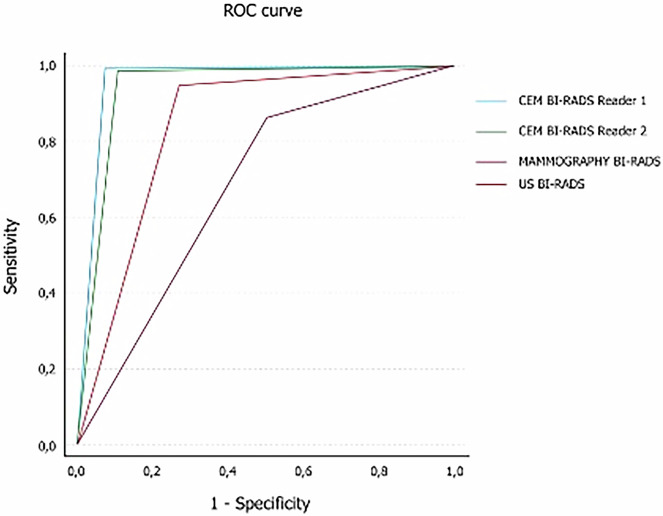
ROC curve comparing CEM, DM, and US examinations

The diagnostic performance of CEM compared with DM and US is shown in Table 3. The sensitivity of CEM for breast cancer detection was > 95% for both Readers, while specificity was approximately 90% for Reader 1 and 85% for Reader 2. In comparison, the sensitivity and specificity of US were 94.6% and 71.4%, respectively, while those of DM were 84.6% and 46.4%, respectively. As a consequence, the overall accuracy of CEM was higher for both readers (94.6% and 92.7% for readers 1 and 2, respectively) when compared with the overall accuracy of US (88.3%) and DM (74.1%). Likewise, PPV and NPV were also markedly higher for CEM (Table 3). The superior diagnostic performance of CEM was confirmed by ROC analysis (Fig. 5). The AUC for CEM Readers 1 and 2 was 0.92 and 0.90, respectively, compared with 0.83 for US and 0.65 for DM.

**Table 3:** Diagnostic performance of mammography, US, and CEM

	Mammography	US	CEM Reader 1	CEM Reader 2
Sensitivity	84.6	94.6	97.3	96.0
Specificity	46.4	71.4	87.5	83.9
PPV	80.8	89.8	95.4	94.1
NPV	53.1	83.3	92.5	88.7
Accuracy	74.1	88.3	94.6	92.7

Similar findings were obtained when the determinations of diagnostic performance were made based on breast composition (Table 4) and in terms of sensitivity for lesion diagnosis by histologic subtype (Table 5). Excellent diagnostic performance was achieved with CEM both in women with dense breast parenchyma and in women with non-dense breast parenchyma.

**Table 4:** Diagnostic performance according to breast composition

Reader 1					Reader 2				
ACR category	Diagnostic performance parameter	Mammo	US	CEM	ACR category	Diagnostic performance parameter	Mammo	US	CEM
A–B (99/205)	Sensitivity	91.9	95.9	95.9	A–B (98/205)	Sensitivity	91.9	94.6	94.6
	Specificity	44	72	84		Specificity	45.8	70.8	79.2
	PPV	82.9	91	94.7		PPV	84	90.9	93.3
	NPV	64.7	85.7	87.5		NPV	64.7	81	82.6
	Accuracy	79.8	89.9	92.9		Accuracy	80.6	88.8	90.9
C–D (106/205)	Sensitivity	77.3	93.3	98.7	C–D (107/205)	Sensitivity	77.3	94.7	97.3
	Specificity	48.4	71	90.3		Specificity	46.9	71.9	87.5
	PPV	78.4	88.6	96.1		PPV	77.3	88.8	94.8
	NPV	46.9	81.5	96.6		NPV	46.9	85.2	93.3
	Accuracy	68.9	86.8	96.2		Accuracy	68.2	87.8	94.4

**Table 5:** Sensitivity for lesion diagnosis according to histologic subtype

	Mammography	US	CEM Reader 1	CEM Reader 2
NST	85.1	96.5	96.5	94.7
ILC	90.5	100	100	100

Agreement between the two readers, as measured using Cohen’s Kappa statistic, was substantial, indicating excellent consistency in image assessment. Cohen’s Kappa values of 0.91, 0.91, and 0.95 were obtained for the determination of breast composition, BPE, and BI-RADS category attribution at CEM, respectively.

Finally, Spearman’s correlation analysis revealed a significant association between BPE and breast composition for both readers: Reader 1 had a ρ of 0.32 (*p* < 0.001), and Reader 2 had a ρ of 0.28 (*p* < 0.001).

## Discussion

Despite the commercial introduction of CEM in 2011, there is still wide variation among practitioners regarding the most suitable acquisition protocol, and very little focus on the most appropriate concentration of CM to use. Typically, it is stated that administered CM should have an iodine concentration of 300–370 mgI/mL and should be administered by power injection at a dose of 1.5 mL/kg (up to a maximum of 150 mL) and at a rate of 2–3 mL/s [2, 3]. However, neither dose-finding studies nor studies that compare different iodine concentrations and flow rates have yet been performed. Moreover, these recommendations lead to a wide variation in the amount of iodine administered, which is potentially concerning in an era of increased focus on sustainable solutions to CM use.

Our study confirms that excellent diagnostic performance is achieved when performing CEM with an iodinated CM containing the highest concentration of iodine currently available (400 mgI/mL). Two blinded readers determined sensitivity and specificity values of 96–97% and 84–87.5%, respectively, and an overall diagnostic accuracy of 93–95%, for the characterization of breast lesions in women referred for CEM as a second-level diagnostic examination. Inter-reader agreement assessed using Cohen’s kappa statistics indicated substantial agreement between readers (Kappa = 0.95 for BI-RADS category attribution), further supporting the consistency of findings. ROC analysis confirmed the good diagnostic performance of CEM with AUC values of 0.90–0.92 for the two blinded readers. Our results are consistent with those of a recent meta-analysis based on 60 studies that reported sensitivity and specificity values of 95% and 81%, respectively, and an overall AUC of 0.94 [5].

Although no data regarding CM type or concentration were included in the abovementioned meta-analysis [5], a systematic review published in 2019 described the contrast administration details of 84 CEM studies performed across 22 countries [14]. Contrast type was reported in 79 (94%) studies, with a 300 mgI/mL concentration used in 34 studies, a 350 mgI/mL concentration in 27 studies, and a 370 mgI/mL concentration in 15 studies. The remaining 3 studies utilized a CM with an iodine concentration of 320 mgI/mL (*n* = 1) or 400 mgI/mL (*n* = 2), the latter in a total of just 26 patients. Full administration details, including dose and injection flow rate, were available for just 69 (82%) studies. Among these studies, the administration of CM at 1.5 mL/kg and at a rate of 3 mL/s was by far the most common practice, utilized in 59 of the 69 studies (at a concentration of 300 mgI/mL in 25 studies, 350 mgI/mL in 23 studies, and 370 mgI/mL in the remaining 11 studies). For a “standard” patient of 75 kg, these injection parameters correspond to 112.5 mL of CM injected over 37.5 s, which results in an IDR of 0.9 g/s (33.75 gI/37.5 s) for patients receiving a CM concentration of 300 mgI/mL, 1.05 g/s (39.375 gI/37.5 s) for patients receiving a CM concentration of 350 mgI/mL, and 1.11 g/s (41.625 gI/37.5 s) for patients receiving a CM concentration of 370 mgI/mL. In our study, a CM containing 400 mgI/mL was administered at 1.0 mL/kg and at a flow rate of 3 mL/s. This corresponds to 75 mL for a 75 kg patient, administered over 25 s, giving an IDR of 1.2 g/s (30 gI/25 s). Clearly, this higher IDR will result in a greater enhancement in the vessels of interest, which is highly desirable for the improved early detection of malignant neoangiogenesis and the identification and diagnosis of fast-growing aggressive lesions with high proliferative and metastatic potential. Moreover, the lower volume administered while maintaining the same injection rate results in a shorter overall injection time and thus greater iodine saving. To this end, our injection of a CM containing 400 mgI/mL at 1.0 mL/kg (i.e., 400 mgI/kg bodyweight) would correspond to 30 g iodine for a “standard” 75 kg patient. This is considerably lower than the total iodine load administered if CM containing 300–370 mg/mL is injected at 1.5 mL/kg (450–555 mgI/kg bodyweight, corresponding to total iodine loads of 33.75–41.63 g iodine for a 75 kg patient).

Not unexpectedly, CEM outperformed conventional DM and US in our study, both in women with dense and non-dense breast parenchyma. This is consistent with previous studies in which other CMs were used [28]. Also consistent with recent findings [29, 30] was a correlation between BPE and breast density in our study. Interestingly, the vast majority of patients in our study had little or no BPE. Although this precluded more detailed analysis, it suggests that the higher iodine concentration of the CM used (400 mgI/mL) and higher IDR do not elicit higher levels of BPE in CEM examinations. In comparison, BPE levels in breast MRI have been shown to be related to the injection rate of the contrast agent [31]. In the case of CEM, higher BPE levels could reflect higher volumes of CM injected over longer injection times, although this remains to be investigated.

Regarding the analysis of the diagnostic performance by lesion type, CEM was markedly superior to conventional DM in terms of sensitivity for the detection of DCIS and invasive breast cancer, achieving a sensitivity of 100% for the detection of DCIS and ILC, and 95–96% for NST. The increase in sensitivity compared to DM, particularly in the case of DCIS, underlines the value of CEM in combining low-energy images showing calcifications and recombined images for lesions characterized by non-mass enhancement or inconsistent enhancement [32]. In this regard, CEM may be superior to breast MRI, allowing the contemporaneous evaluation of calcifications and enhancement [33].

Several authors have shown previously that CEM, like MRI, is effective at distinguishing among breast cancer molecular subtypes [34–36]. Our study, likewise, has shown that CEM with HCCM is superior to conventional DM in differentiating molecular subtypes, in particular for HER2-positive and luminal-like lesions. Although these preliminary findings need to be confirmed in larger cohorts of patients, it is plausible that the simultaneous evaluation of both calcifications and enhancement on CEM is particularly advantageous for distinguishing certain histologic subtypes given that different patterns of calcifications are known to be predicable of HER2-positive and luminal A breast cancer [37–39].

Our study has several limitations. Firstly, it was retrospective in nature. Secondly, the patient population was relatively limited, even though our population was wider and with a greater range of breast lesions than many other studies on CEM [5, 14]. Thirdly, we did not fully explore potential variations in CEM effectiveness based on patient factors such as body mass index (BMI) or renal function. This was beyond the scope of this initial exploratory study, but should certainly be addressed in subsequent studies. Although patients with impaired renal function were excluded from this study, it should be borne in mind that a CEM protocol that requires lower CM volumes and lower overall iodine loads is likely to be beneficial, particularly in patients with borderline renal insufficiency [21, 40]. Finally, we did not compare different CM concentrations and injection protocols, and thus it was not possible to directly assert the superiority of HCCM compared with other CM concentrations in terms of diagnostic performance. On the other hand, based on the available literature, we can affirm that similar diagnostic performance can be achieved with HCCM at a lower overall injected volume (1.0 mL/kg) and iodine load. The proposed method aligns with current attempts to improve long-term sustainability in iodinated CM usage and with concerns over iodinated CM release into the environment [15, 40–42]. This approach not only reduces costs and resource consumption but also minimizes patient exposure, promoting a more sustainable and patient-friendly strategy for breast cancer screening.

In conclusion, our two-center retrospective study demonstrates excellent diagnostic performance of CEM using HCCM (400 mgI/mL). Benefits of HCCM for CEM include a higher achievable IDR, a lower overall iodine dose, and a shorter injection time. Furthermore. The multicenter, multivendor nature of our research ensures the robustness of our findings. Taken together, our results suggest that HCCM may offer a more sustainable approach to the use of iodinated CM in CEM without loss of diagnostic performance.

## Data Availability

The database is available to any author who requires further verification, suitable for motivation.

## Abbreviations

AUC: Area under the curve
BI-RADS: Breast imaging reporting and data system
BPE: Background parenchymal enhancement
CEM: Contrast-enhanced mammography
CM: Contrast medium
CNB: Core needle biopsy
DCIS: Ductal carcinoma in situ
DM: Digital mammography
HCCM: High concentration contrast medium
IDR: Iodine delivery rate
ILC: Invasive lobular carcinoma
MRI: Magnetic resonance imaging
NST: Invasive cancer of no special type
NPV: Negative predictive value
PPV: Positive predictive value
ROC: Receiver operating characteristic
US: Ultrasound
VAB: Vacuum-assisted biopsy

## References

[1] Cozzi A, Schiaffino S, Sardanelli F (2019) The emerging role of contrast-enhanced mammography. Quant Imaging Med Surg 9:2012–2018 31929976 10.21037/qims.2019.11.09 PMC6942965

[2] Jochelson MS, Lobbes MBI (2021) Contrast-enhanced mammography: state of the art. Radiology 299:36–48 33650905 10.1148/radiol.2021201948 PMC7997616

[3] Zamora K, Allen E, Hermecz B (2021) Contrast mammography in clinical practice: current uses and potential diagnostic dilemmas. Clin Imaging 71:126–135 33197726 10.1016/j.clinimag.2020.11.002

[4] Sogani J, Mango VL, Keating D, Sung JS, Jochelson MS (2021) Contrast-enhanced mammography: past, present, and future. Clin Imaging 69:269–279 33032103 10.1016/j.clinimag.2020.09.003 PMC8494428

[5] Cozzi A, Magni V, Zanardo M, Schiaffino S, Sardanelli F (2022) Contrast-enhanced mammography: a systematic review and meta-analysis of diagnostic performance. Radiology 302:568–581 34904875 10.1148/radiol.211412

[6] Neeter LMFH, Raat HPJF, Alcantara R et al (2021) Contrast-enhanced mammography: what the radiologist needs to know. BJR Open 3:20210034 34877457 10.1259/bjro.20210034 PMC8611680

[7] Clauser P, Baltzer PAT, Kapetas P et al (2020) Low-dose, contrast-enhanced mammography compared to contrast-enhanced breast MRI: a feasibility study. J Magn Reson Imaging 52:589–595 32061002 10.1002/jmri.27079 PMC7496227

[8] Fallenberg EM, Schmitzberger FF, Amer H et al (2017) Contrast-enhanced spectral mammography vs. mammography and MRI—clinical performance in a multi-reader evaluation. Eur Radiol 27:2752–2764 27896471 10.1007/s00330-016-4650-6

[9] Hobbs MM, Taylor DB, Buzynski S, Peake RE (2015) Contrast-enhanced spectral mammography (CESM) and contrast-enhanced MRI (CEMRI): patient preferences and tolerance. J Med Imaging Radiat Oncol 59:300–305 25900704 10.1111/1754-9485.12296

[10] Neeter LMFH, Robbe MMQ, van Nijnatten TJA et al (2023) Comparing the diagnostic performance of contrast-enhanced mammography and breast MRI: a systematic review and meta-analysis. J Cancer 14:174–182 36605487 10.7150/jca.79747 PMC9809339

[11] Lobbes MBI, Neeter LMFH, Raat F et al (2023) The performance of contrast-enhanced mammography and breast MRI in local preoperative staging of invasive lobular breast cancer. Eur J Radiol 164:110881 37201248 10.1016/j.ejrad.2023.110881

[12] Lobbes MBI, Jochelson MS, Neeter LMFH, Nelemans PJ (2023) Contrast-enhanced mammography and breast MRI: Friends or foes? Radiology 307:e221558 10.1148/radiol.221558 36413132

[13] Pötsch N, Vatteroni G, Clauser P, Helbich TH, Baltzer PAT (2022) Contrast-enhanced mammography versus contrast-enhanced breast MRI: a systematic review and meta-analysis. Radiology 305:94–103 36154284 10.1148/radiol.212530

[14] Zanardo M, Cozzi A, Trimboli RM et al (2019) Technique, protocols and adverse reactions for contrast-enhanced spectral mammography (CESM): a systematic review. Insights Imaging 10:76 31376021 10.1186/s13244-019-0756-0 PMC6677840

[15] Koeppel DR, Boehm IB (2023) Shortage of iodinated contrast media: status and possible chances—a systematic review. Eur J Radiol 164:110853 37156181 10.1016/j.ejrad.2023.110853 PMC10155429

[16] Kaiser DPO, Abdalkader M, Berberich A, Sporns PB, Nguyen TN (2022) Acute shortage of iodinated contrast media: implications and guidance for neurovascular imaging and intervention. Neuroradiology 64:1715–1718 35716206 10.1007/s00234-022-02999-6 PMC9206091

[17] Dekker HM, Stroomberg GJ, Prokop M (2022) Tackling the increasing contamination of the water supply by iodinated contrast media. Insights Imaging 13:30 35201493 10.1186/s13244-022-01175-x PMC8873335

[18] Jeong CH, Machek EJ, Shakeri M et al (2017) The impact of iodinated X-ray contrast agents on formation and toxicity of disinfection by-products in drinking water. J Environ Sci 58:173–182 10.1016/j.jes.2017.03.032 28774606

[19] England A, Rawashdeh M, Moore N et al (2024) More sustainable use of iodinated contrast media: Why? Radiography 30:74–80 38991461 10.1016/j.radi.2024.06.023

[20] Sun YX, Shang J, Yong C et al (2024) Impact of different concentration iodinated contrast media on pain and comfort in abdominal computed tomography. Eur J Radiol 179:111664 39121745 10.1016/j.ejrad.2024.111664

[21] Thomsen HS, Morcos SK, Erley CM et al (2008) The ACTIVE Trial: comparison of the effects on renal function of iomeprol-400 and iodixanol-320 in patients with chronic kidney disease undergoing abdominal computed tomography. Invest Radiol 43:170–178 18301313 10.1097/RLI.0b013e31815f3172

[22] ESUR. ESUR guidelines on contrast agents. European Society of Urogenital Radiology. Available via https://www.esur.org/esur-guidelines-on-contrast-agents/. Accessed 27 Sep 2024

[23] D’Orsi CJ, Sickles EA, Mendelson EB, Morris EA et al (2013) ACR BI-RADS® atlas, breast imaging reporting and data system. American College of Radiology, Reston

[24] Janice SS, John ML, Jordana P et al (2022) Contrast enhanced mammography (CEM) (a supplement to ACR BI-RADS® Mammography 2013). ACR BI-RADS® atlas, breast imaging reporting and data system. American College of Radiology, Reston

[25] Lakhani S, Ellis I, Schnitt S et al (2012) WHO classification of tumours of the breast, 4th edn. IARC Press, Lyon

[26] Wolff AC, Hammond MEH, Hicks DG et al (2014) Recommendations for human epidermal growth factor receptor 2 testing in breast cancer: American Society of Clinical Oncology/College of American Pathologists clinical practice guideline update. Arch Pathol Lab Med 138:241–256. 10.5858/arpa.2013-0953-SA 24099077 10.5858/arpa.2013-0953-SA PMC4086638

[27] Goldhirsch A, Winer EP, Coates AS et al (2013) Personalizing the treatment of women with early breast cancer: highlights of the St Gallen International Expert Consensus on the Primary Therapy of Early Breast Cancer. Ann Oncol 24:2206–2223. 10.1093/annonc/mdt303 23917950 10.1093/annonc/mdt303 PMC3755334

[28] Moffa G, Galati F, Maroncelli R et al (2023) Diagnostic performance of contrast-enhanced digital mammography versus conventional imaging in women with dense breasts. Diagnostics (Basel) 13:2520. 10.3390/diagnostics13152520 37568883 10.3390/diagnostics13152520 PMC10416841

[29] Magni V, Cozzi A, Muscogiuri G et al (2024) Background parenchymal enhancement on contrast-enhanced mammography: associations with breast density and patient’s characteristics. Radiol Med 129:1303–1312. 10.1007/s11547-024-01860-5 39060886 10.1007/s11547-024-01860-5

[30] Moffa G, Galati F, Spagnoli A et al (2024) BPE on contrast-enhanced mammography: relationship with breast density, age and menopausal status. Radiol Med. 10.1007/s11547-024-01912-w 10.1007/s11547-024-01912-w 39535654

[31] Marzocca F, Moffa G, Landi VN et al (2022) Gadoteridol-enhanced MRI of the breast: Can contrast agent injection rate impact background parenchymal enhancement? Acta Radiol 63:1173–1179. 10.1177/02841851211034038 34323589 10.1177/02841851211034038

[32] Elder K, Matheson J, Nickson C et al (2023) Contrast-enhanced mammography in breast cancer surveillance. Breast Cancer Res Treat 199:221–230. 10.1007/s10549-023-06916-0 36966271 10.1007/s10549-023-06916-0 PMC10175447

[33] Taylor DB, Burrows S, Saunders CM et al (2023) Contrast-enhanced mammography (CEM) versus MRI for breast cancer staging: detection of additional malignant lesions not seen on conventional imaging. Eur Radiol Exp 7:8. 10.1186/s41747-022-00318-5 36781808 10.1186/s41747-022-00318-5 PMC9925630

[34] Wang S, Wang Z, Li R et al (2022) Association between quantitative and qualitative image features of contrast-enhanced mammography and molecular subtypes of breast cancer. Quant Imaging Med Surg 12:1270–1280 35111622 10.21037/qims-21-589 PMC8739155

[35] Cheng BW, Ko TY, Lai YTA (2024) Radiologic-pathologic correlation: is there an association between contrast-enhanced mammography imaging features and molecular subtypes of breast cancer? Cureus 16:e64791 10.7759/cureus.64791 PMC11329886 39156463

[36] Li N, Gong W, Xie Y, Sheng L (2023) Correlation between the CEM imaging characteristics and different molecular subtypes of breast cancer. Breast 72:103595 37925875 10.1016/j.breast.2023.103595 PMC10661457

[37] Boisserie-Lacroix M, Bullier B, Hurtevent-Labrot G et al (2014) Correlation between imaging and prognostic factors: molecular classification of breast cancers. Diagn Interv Imaging 95:227–233 24508482 10.1016/j.diii.2013.12.013

[38] Cen D, Xu L, Li N et al (2017) BI-RADS 3–5 microcalcifications can preoperatively predict breast cancer HER2 and luminal A molecular subtype. Oncotarget 8:13855–13862 28099938 10.18632/oncotarget.14655 PMC5355144

[39] Tan PS, Ali MA, Eriksson M et al (2021) Mammography features for early markers of aggressive breast cancer subtypes and tumor characteristics: a population-based cohort study. Int J Cancer 148:1351–1359 32976625 10.1002/ijc.33309 PMC7891615

[40] McCullough PA, Stacul F, Becker CR et al (2006) Contrast-induced nephropathy (CIN) consensus working panel: executive summary. Rev Cardiovasc Med 7:177–197 17224862

[41] England A, Rawashdeh M, Moore N, Young R, Curran G, McEntee MF (2024) More sustainable use of iodinated contrast media—Why? Radiography 30:74–80 38991461 10.1016/j.radi.2024.06.023

[42] Beer M, Schuler J, Kraus E et al (2023) Discharge of iodine-containing contrast media into the environment—problem analysis and implementation of measures to reduce discharge by means of separation toilets—experience from a pilot project. Rofo 195:1122–1127 37793416 10.1055/a-2168-8346

